# Comparing the biomechanical properties of conventional suture and all-suture anchors using patient-specific and realistic osteoporotic and non-osteoporotic phantom using 3D printing

**DOI:** 10.1038/s41598-023-48392-y

**Published:** 2023-11-28

**Authors:** Junhyeok Ock, Junghyun seo, Kyoung Hwan Koh, Namkug Kim

**Affiliations:** 1 Department of Biomedical Engineering, Asan Medical Center, Asan Medical Institute of Convergence Science and Technology, University of Ulsan College of Medicine, Pungnap2-dong, Songpa-gu, Seoul, South Korea; 2 Department of Orthopaedic Surgery, Asan Medical Center, University of Ulsan College of Medicine, 88 Olympic-Ro 43-Gil Songpa-Gu, Seoul, 05505 South Korea; 3 Department of Radiology, Asan Medical Center, University of Ulsan College of Medicine, 388-1 Pungnap2-dong, Songpa-gu, Seoul, South Korea; 4 Department of Convergence Medicine, Asan Medical Center, Asan Medical Institute of Convergence Science and Technology, University of Ulsan College of Medicine, 88 Olympic-Ro 43-Gil Songpa-Gu, Seoul, 05505 South Korea

**Keywords:** Biomedical engineering, Bone imaging, Musculoskeletal system

## Abstract

Conventional suture anchors (CAs) and all-suture anchors (ASAs) are used for rotator cuff repair. Pull-out strength (POS) is an important factor that affects surgical outcomes. While the fixation mechanism differs between the anchor types and relies on the quality, few studies have compared biomechanical properties of anchors based on bone quality. This study aimed to compare the biomechanical properties of anchors using osteoporotic bone (OB) and non-osteoporotic bone (NOB) simulators. Humerus simulators were fabricated using fused deposition modeling of 3D printing and acrylonitrile butadiene styrene adjusting the thickness of cortical bone and density of cancellous bone based on CT images. Cyclic loading from 10 to 50 N, 10 to 100 N, and 10 to 150 N for 10 cycles was clinically determined at each anchor because the supraspinatus generates a force of 67–125 N in daily activities of normal control. After cyclic loading, the anchor was extruded at a load of 5 mm/min. Displacement, POS, and stiffness were measured. In OB simulators, CAs revealed bigger gap displacement than ASAs with cyclic loading of 10–150 N. ASA showed higher values for POS and stiffness. In NOB simulators, ASAs revealed bigger gap displacement than CAs with cyclic loading of 10–150 N. ASA showed higher values for POS and CA showed higher values for stiffness. POS of anchors depends on anchors ‘displacement and bone stiffness. In conclusion, ASA demonstrated better biomechanical performance than CA in terms of stability under cyclic loading and stiffness with similar POS in OB.

## Introduction

Rotator cuff repair surgery is a transosseous equivalent repair that is performed using a suture anchor similar to that in conventional transosseous repair^1^. Currently, screw-in type of conventional suture anchors (CAs) and all-suture anchors (ASAs) have become popular in rotator cuff repair surgery^1–3^. Most repairs performed using CAs provide sufficient fixation force until tendon-to-bone healing is achieved; however, this depends on the bone quality^4^. In contrast, ASAs depend on the quality of the cortical bone rather than that of the cancellous bone^5^. The pull-out strength (POS) of a suture anchor is one of the determining factors of early failure. Therefore, the POS of ASAs and CAs has been evaluated in various bone quality environments^1–5^. Furthermore, biomechanical tests have been conducted using human cadaveric specimens^6–8^. To thoroughly understand the characteristics of the anchor, specimens that reflect the mechanical properties of osteoporotic bone (OB) and non-osteoporotic bone (NOB) should be used^5^. However, cadaveric bone is difficult to obtain and has ethical issues. Furthermore, the shape, bone quality, and strength of bones vary between cadavers. Consequently, the standard deviation could become exceptionally large under similar experimental conditions^9^. Due to these difficulties, recent studies have performed biomechanical tests using synthetic plastic bone^7,10^. Synthetic bones have uniform mechanical properties and can be obtained easily, although they do not precisely reflect the mechanical properties of OBs and NOBs^11–13^. We propose the development of patient-specific and realistic humerus simulators for anchor insertion using three-dimensional (3D) printing and deriving the mechanical properties using computed tomography (CT). The purpose of this study was to evaluate and compare the biomechanical properties of CAs and ASAs in 3D-printed OB and NOB simulators. We aimed to verify the hypothesis that ASAs are stronger than CAs in OBs.

## Methods

For fabrication and evaluation of the humerus bone simulator, the bone was segmented and designed for biomechanical testing based on the CT images. Then, the simulators were fabricated using a 3D printer with the appropriate mechanical properties. Biomechanical testing was performed by extruding the anchors inserted into these fabricated simulators.

### Preparation of the humerus simulator

**Figure 1:**
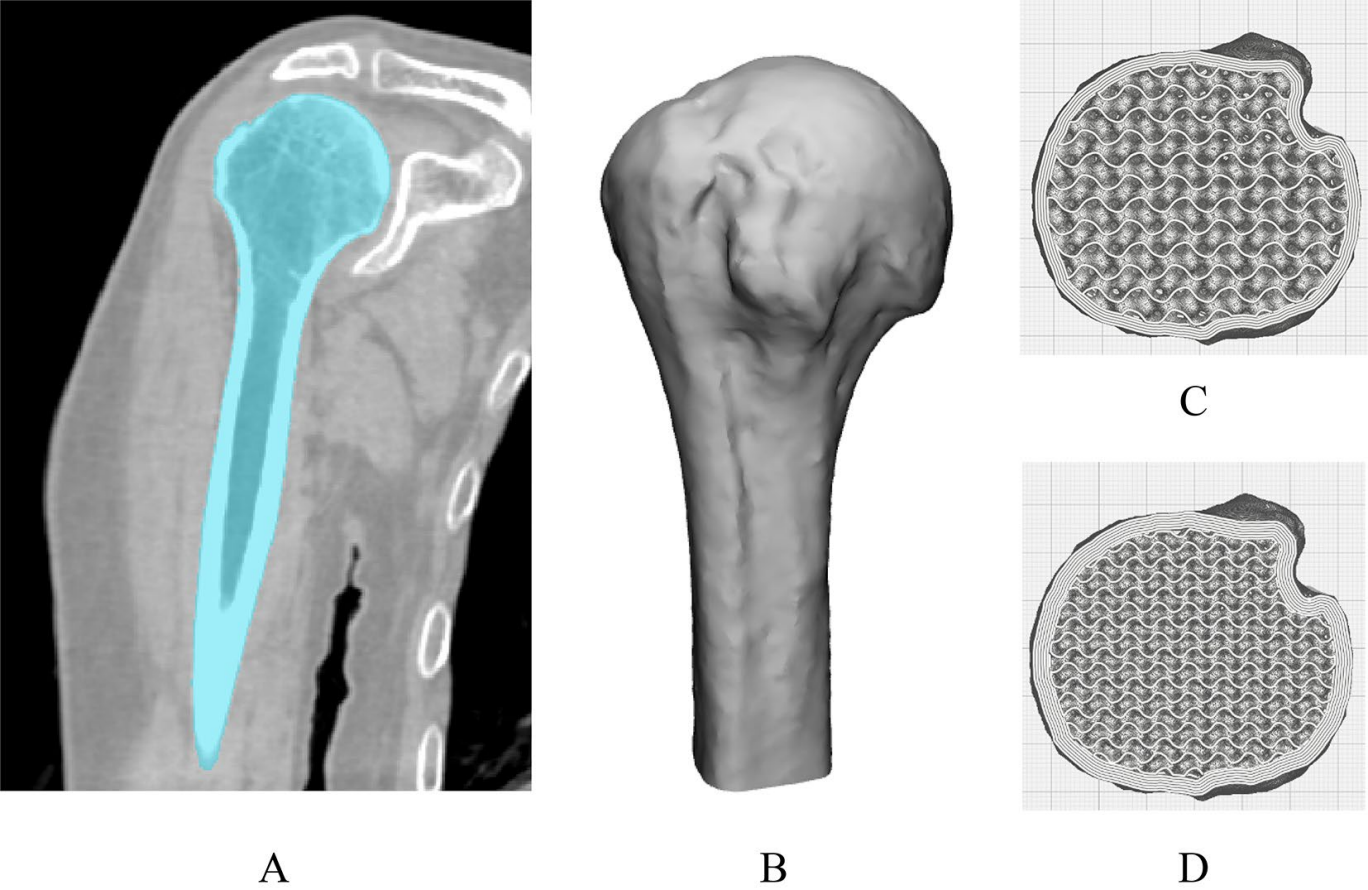
Visualization of segmentation of the humerus and the humerus simulator based on computed tomography (CT) in a patient requiring shoulder joint replacement surgery. (**A**) Coronal view of the humerus on CT. (**B**) Three-dimensional (3D) visualized humerus model. (**C**) Top view of the osteoporotic bone simulator with the internal pattern (**D**) Top view of the non-osteoporotic bone simulator with the internal pattern.

**Figure 2:**
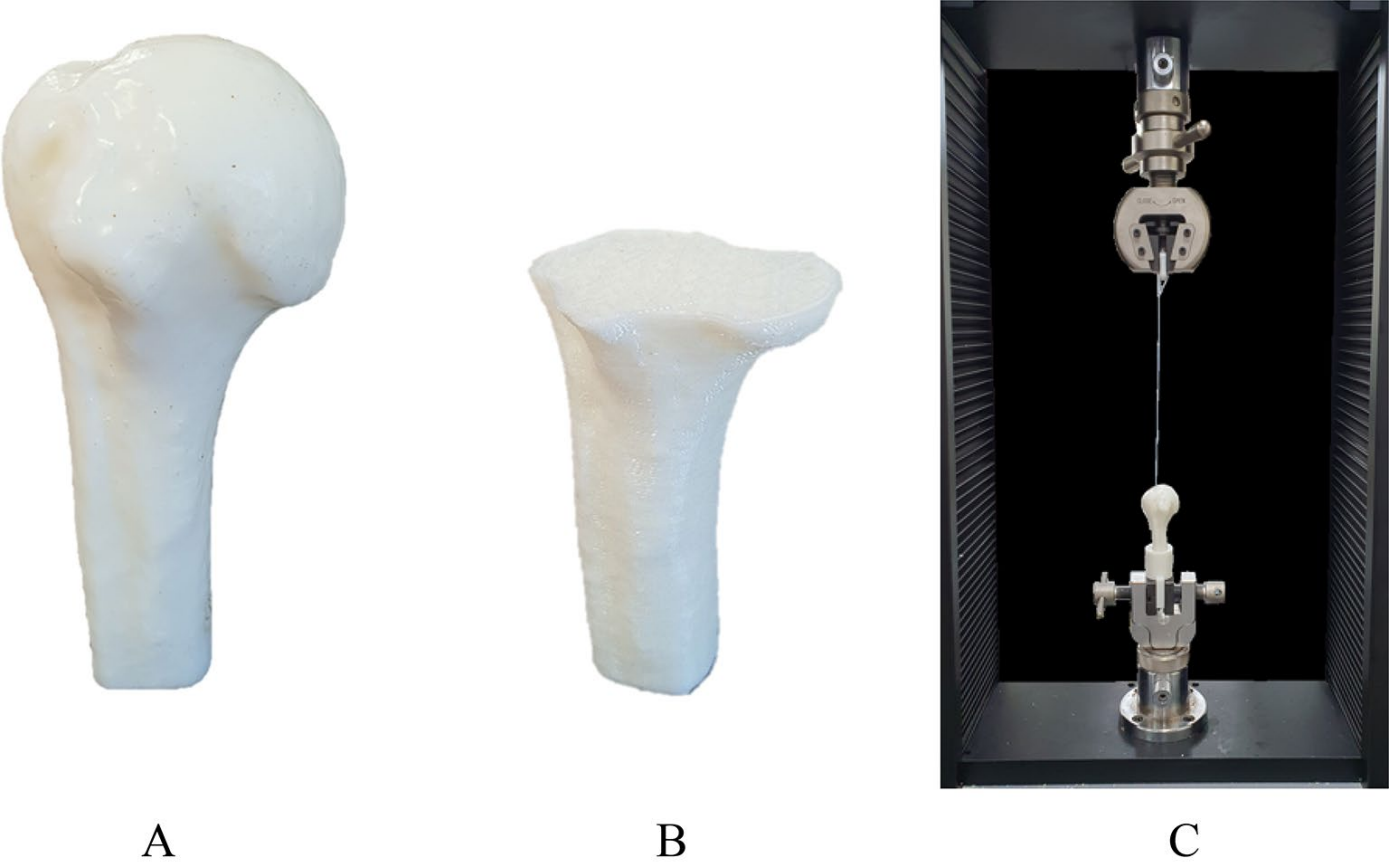
Fabricated humerus simulator and the experiment settings. (**A**) Fabricated humerus specimens. (**B**) Visualization of the humerus along with the internal pattern. (**C**) Experiment settings for biomechanical testing.

This study was approved by the Institutional Review Board of Asan Medical Center (IRB No. 2021-2046) and was performed according to the principles of the Declaration of Helsinki. The requirement for informed consent was waived by the Institutional Review Board due to the retrospective observational design. Anonymized multiple detector CT images by removing personal information were obtained using a 120-Kvp tube voltage and 1-mm slice thickness in a 71-year-old male who was scheduled for shoulder joint replacement surgery. The humerus was segmented using the thresholding function of 123–1546 Hounsfield units as well as the region growing functions from manually chosen seeds by an expert (Fig. 1A). The segmented humerus was converted to stereolithography format. As all areas of the humerus are not required to measure the POS, only an area of 100 mm from the top was used as the region of interest (Fig. 1A). In patients in their 60 s, the density of the greater tuberosity (GT) in the shoulder with rotator cuff tears is 200 mg/cm^3^, while that on the healthy side is 300 mg/cm^3^^14^. Furthermore, the cortical thickness of the proximal humerus is approximately 2 mm^15^. Therefore, The OB simulator was designed with an outer layer of 1.5 mm by reducing the thickness by 0.5 mm, the internal structure was designed with a gyroid shape infill to 15% to create a density of 200 mg/cm^3^. The NOB simulator was designed with an outer layer of 2.5 mm by increasing the thickness by 0.5 mm, the internal structure was designed with a gyroid shape infill to 22% to create a density of 300 mg/cm^3^. Two patterns are visualzied in Fig. 1C,D. The mean compressive strength for the human humerus is 107 MPa and a 3D-printed acrylonitrile butadiene styrene (ABS) specimens can have a compressive strength of approximately 100 MPa following surface treatment^16,17^. Therefore, the simulators were fabricated using fused deposition modeling (FDM) with ABS filament (Ultimaker S5, Ultimaker, The Netherlands), and the surface was polished using fumigation in a vapor treatment machine (Bboshasi 250k, Kobot, South Korea) for 7 min. Finally, 20 artificial proximal humerus simulators (10 OB and 10 NOB stimulators) were fabricated (Fig. 2A,B).

### Preparation of the suture anchors and implantation

Commercially available Q-Fix (outer diameter 2.8 mm, length 20 mm) ASAs (Smith & Nephew, Andover, MA, USA) and Healicoil (outer diameter 5.5 mm, length 18 mm) CAs (Smith & Nephew) were used. Q-fix expands to approximately 5.5 mm after deployment, and the maximum outer diameter of Healicoil was 5.5 mm. As their scalability is similar, we used both anchors to evaluate if there was any difference between the anchor types due to bone quality. The orthopedic surgeon inserted both anchors according to the manufacturer's instructions. A pilot hole tapping guide was placed on the GT of each humerus simulator and the pilot hole size of ASAs and CAs a diameter of 1 mm and 3 mm pilot hole was made using an awl. Each anchor was inserted anchors vertically into the GT of the humerus simulator through the guide, it was firmly fixed using a mallet. Subsequently, guide, handle, and suture strands were removed, sequentially. Suture strands and the body of the humerus were fixed on a universal testing machine (ST-1001, SALT Corp., Korea). A total of 20 biomechanical tests were determined through the Design of Experiments (DoE) via Taguchi methods was designated using 2 parameters, 2 levels, and 5 repeats (Table 1). The number of repetitions of each condition was determined through the TTestIndPower package in Pythons (effect size = 0.8, alpha error = 0.05, power = 0.2). Five experiments were conducted for each condition (5 CA with OB, 5 CA with NOB, 5 ASA with OB, and 5 ASA with NOB), and a total of 20 biomechanical tests were conducted^18^.

**Table 1:** Parameters and levels of design of experiments.

Parameter	Level 1	Level 2
All-suture anchor	Osteoporotic bone simulator	Non-osteoporotic bone simulator
Conventional suture anchor	Osteoporotic bone simulator	Non-osteoporotic bone simulator

### Biomechanical testing

POS, stiffness, and gap displacement under cyclic loading were measured using a universal testing machine with a 500-N load cell and at room temperature (Fig. 2C). We applied a preload of 10 N in 1 min to the anchor via the sutures prior to cyclic loading over the inserted anchor. The supraspinatus generates a force of 67–125 N in daily activities^19^. Patients may engage in activities cautiously but may inevitably make large movements. Cyclic loading from 10 to 50 N, 10 to 100 N, and 10 to 150 N for 10 cycles each at a rate of 0.5 Hz was clinically determined by an expert orthopedic surgeon with more than 20 years of experience based on daily supraspinatus generates a force. The gap displacement of the extruded anchor was measured using vernier calipers with repeat accuracy of 0.01 mm (CD-30AX, Mitutoyo Co., Japan) at the end of each cycle. Subsequently, we measured the POS and stiffness by extruding the anchor at a speed of 5 mm/min according to American Society for Testing and Materials F1839-08. The test of the above conditions was continually conducted on one simulator in reuse manner.

### Statistical analysis

The difference in the POS and stiffness between ASAs and CAs in OBs and NOBs were analyzed using the Mann–Whitney U test. The pattern between the ASA and CA in terms of the gap displacement with accumulated cyclic loading was compared using the Friedman test. Finally, the groups were compared individually using the Mann–Whitney U test. The significance level was set to *p* < 0.05, and the analyses were performed using Excel (Microsoft Inc., Redmond, WA, USA) and SPSS v25 (IBM Corp., Armonk, NY, USA).

## Results

### Biomechanical testing

**Figure 3:**
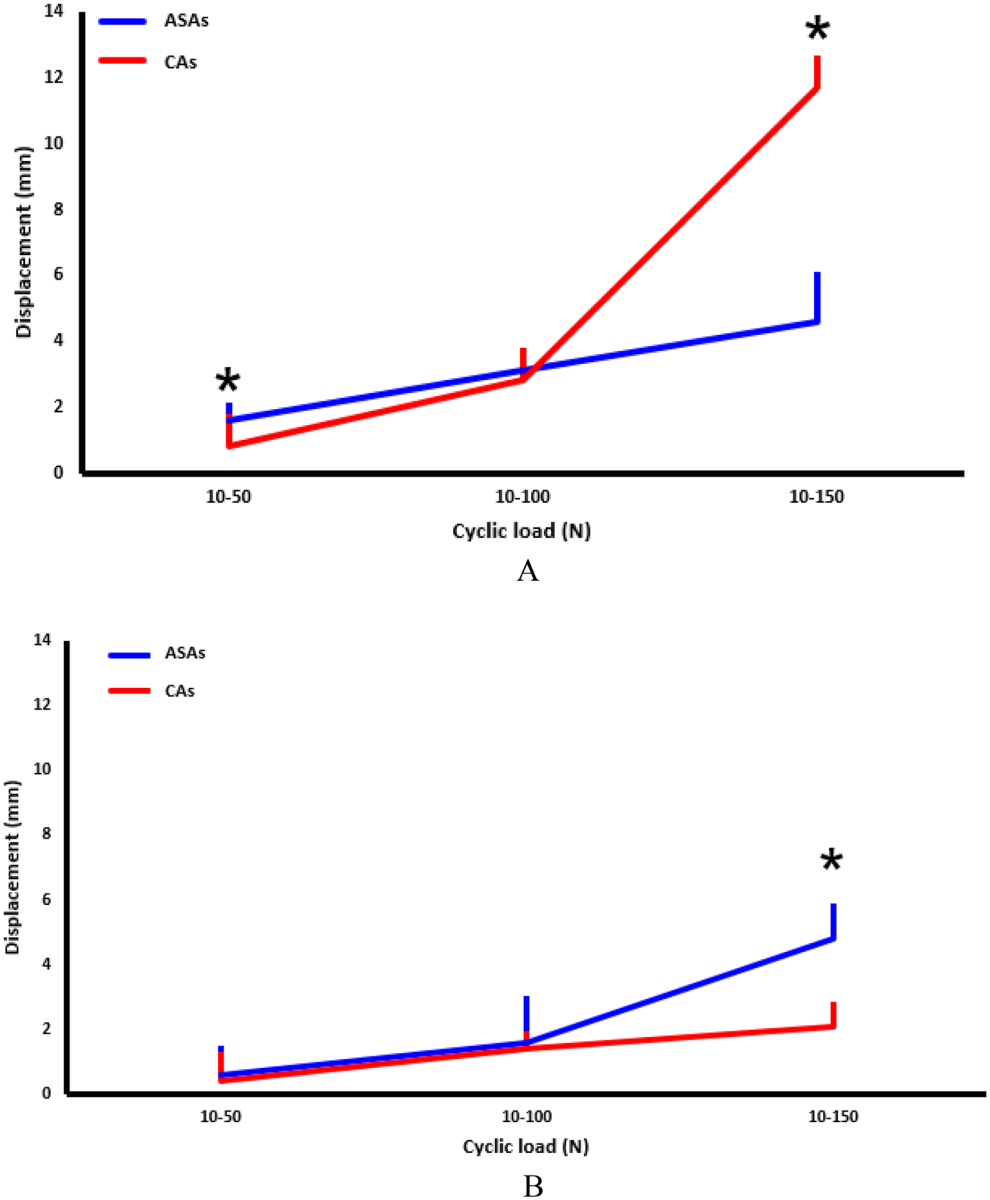
Gap displacement of CAs and ASAs in OBs and NOBs with cyclic loads of 10–50, 10–100, and 10–150 N. (**A**) In OBs, the gap displacement pattern varied between ASAs (red) and CAs (blue) (*p* < 0.001). Cyclic loading of 10–50 N (1.6 vs. 0.8 mm, respectively, *p* = 0.037) and 10–150 N (4.6 vs. 11.7 mm, *p* = 0.009) revealed significant differences between the two types of anchors. (**B**) In NOBs, ASAs and CAs also revealed different patterns of gap displacement with cyclic loading (*p* = 0.001). The displacement with 10–150 N of cyclic loading was higher in ASAs than that in CAs (4.8 vs 2.1 mm, respectively, *p* = 0.011). CA: conventional anchor (Healicoil (5.5 mm), Smith & Nephew, Andover, MA, USA), ASA: all-suture anchor (Q-Fix (2.8 mm), Smith & Nephew, Andover, MA, USA), OB: osteoporotic bone, NOB: non-osteoporosis bone.

**Figure 4:**
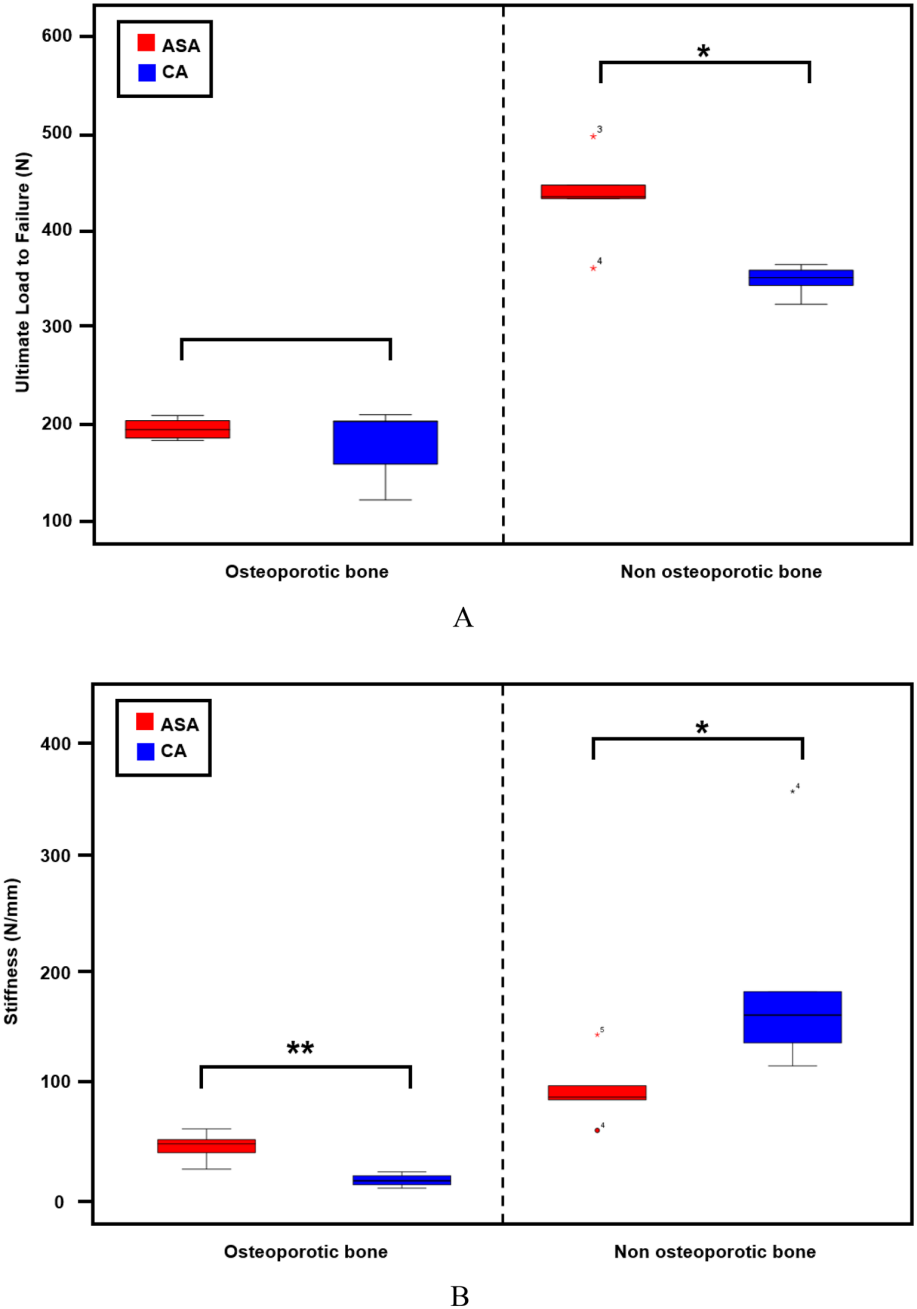
Box plot of load to failure and stiffness of each test group in osteoporotic bone and non-osteoporotic bone using all-suture anchors (Q-Fix (2.8 mm), Smith & Nephew, Andover, MA, USA) (red) and conventional anchors (Healicoil (5.5 mm), Smith & Nephew, Andover, MA, USA) (blue). (**A**) The ultimate load to failure. (**B**) Stiffness. *p < 0.05; **p < 0.01.

In OBs with ASAs, the gap displacement (mean ± standard deviation [SD]) with cyclic loading of 10–50, 10–100, and 10–150 N was 1.6 ± 0.5, 3.1 ± 0.41, and 4.6 ± 1.5 mm, respectively (Fig. 3A). In OBs with CAs, the gap displacement was 0.8 ± 0.4, 2.8 ± 0.8, and 11.7 ± 3.4 mm, respectively (Fig. 3A). In OBs, the gap displacement with the accumulation of cyclic loading was significantly different between ASAs and CAs (*p* < 0.001). In OBs with CAs, the gap displacement was significantly different between cyclic loading of 10–50 N (*p* = 0.037) and 10–150 N (*p* = 0.009). In NOBs with ASAs, the gap displacement with cyclic loading of 10–50, 10–100, and 10–150 N was 0.6 ± 0.9, 1.6 ± 1.4, and 4.8 ± 1.1 mm, respectively (Fig. 3B). In NOBs with CAs, the gap displacement was 0.4 ± 0.9, 1.4 ± 0.5, and 2.1 ± 0.7 mm, respectively (Fig. 3B). In NOBs, the gap displacement with the accumulation of cyclic loads was significantly different between ASAs and CAs (*p* = 0.001). Specifically, a significant gap displacement with ASA was identified with 10–150 N of cyclic loading (*p* = 0.011) in NOBs. The POSs (mean ± SD) of ASAs (193.5 ± 11.2 N) and CAs (177.6 ± 38.1 N) were not significantly different in OBs (*p* = 0.841). The POSs of ASAs and CAs were significantly different in NOBs (433.5 ± 48.8 and 347.2 ± 16.0 N, respectively; *p* = 0.015), although both POSs were > 300 N, which is strong enough to be used in rotator cuff repair (Fig. 4A). The stiffness (mean ± SD) of OB with ASAs and CAs was 45.5 ± 13.3 and 16.2 ± 5.8 N/mm, respectively, with a significant difference between them (*p* = 0.007). The stiffness of NOB with ASAs and CAs was 95.8 ± 30.6 and 196.8 ± 96.5 N/mm, respectively, with a significant difference (*p* = 0.031) (Fig. 4B).

## Discussion

The most interesting finding of this study was that CAs in OBs revealed a relatively larger gap displacement after cyclic loading of 10–150 N than ASAs, while ASAs revealed a greater gap displacement than CAs in NOBs. It might imply that CA is weaker in OBs with higher cyclic loading, while ASA is weaker in NOBs. In OB, CA shows lower gap displacement than ASA at cyclic loads below 10–100 N, however, the trend reversed at 10–150 N. Therefore, it would be preferable to use CA in patients with low activity and ASA in patients with high activity. In NOB, ASA and CA show similar gap displacement at cyclic loads below 10–100 N, however, ASA higher gap displacement than CA at 10–150 N. Therefore, it would be preferable to use CA in patients with high activity. Optimal healing occurred when the gap between the surgical site and the anchor was less than 5 mm^20^. In NOB, both anchors can expect optimal treatment effects. In OB with CA, it seems difficult to expect optimal treatment effects in patients of high activity. Additionally, while the POS was similar between the anchors in OBs, the stiffness was greater with ASAs than that with CAs, which implied that the strength of the bone to withstand POS was stronger with ASAs in OBs than that with CAs. These findings highlight the overall better biomechanical performance and optimal healing of ASAs over CAs, especially in OBs. Treating patients with osteoporosis and rotator cuff repair using CAs may result in a risk of failure^21^. Therefore, rotator cuff repair using ASAs should be considered for older patients, females, or patients with risk factors for osteoporosis; however, the cortical bone should be preserved. Our results assume an intact cortical bone, although we simulated osteoporosis both in trabecular and cortical bone. Conventionally, the cortical bone is removed to promote healing from the bleeding bone bed in rotator cuff repair. If we damage the cortical bone for bone bed preparation, the fixation strength of ASA is decreased significantly. Some researchers have highlighted the importance of cortical bone preservation to hold ASAs^22^. Therefore, whether the cortex should be preserved for ASAs or should be removed for better healing along with the use of CAs, especially in OBs, warrants further investigations.

In our study, NOBs revealed sufficient POS (433.5 ± 48.8 N for ASA; 347.2 ± 16.0 N for CA) and stiffness (95.8 ± 30.6 N/mm for ASA; 190.8 ± 96.5 N/mm for CA). NOBs revealed < 5 mm gap displacement for cyclic loading with both anchors as well. Although there were statistically significant differences in all parameters, even the weak ASAs revealed enough biomechanical properties to be used in rotator cuff repair of NOBs. Both ASAs and CAs appear to work well in NOBs; however, ASAs are inserted through a 2.8-mm pilot hole, which can minimize bone loss. Therefore, ASAs may be advantageous over CAs in NOBs^5^.

Despite previous studies using synthetic simulators, the understanding of variations in the thickness of the cortical bone (osteoporosis) and its effects on POS remains limited^7,23^. In our study, POS was not significantly different between ASAs and CAs in OBs, as both anchors are primarily influenced by cortex thickness or stiffness. The weak POS following CA in OBs was expected due to trabecular bone quality dependence^4^. While the POS of ASA was similar, the stiffness was greater than that of CA in OBs. Collectively, ASA could be a better alternative than CA in OBs.

We designed OBs and NOBs based on the density of trabecular bone and the thickness of the cortical layer^14,15^. The simulators were fabricated using ABS, whose compressive strength is similar to that of the humerus^16,17^. According to Barber et al., the POS of the synthetic plastic bone and that of porcine bone using Q-Fix 2.8 mm is 494.7 ± 1.1 and 495.1 ± 87.9 N, respectively^24^. In our study, the POS of ASAs in NOBs was 433.5 ± 48.8 N, which was similar to that in the previous study. The POS of CAs in OBs was 177.6 ± 38.1 N, which was similar to the findings of Yamauchi et al. who used Healicoil (5.5 mm) and reported that the POS of synthetic plastic bone with osteoporosis was 146.3 ± 5.8 N^25^. Therefore, our simulator model demonstrated a similar POS as previous studies using polyurethane foam and animal models. The stiffness of both ASA and CA in human humeri aged between 50 and 73 years old is around 70 N/mm^26^. In our study stiffness of the ASA and CA is in NOB 95.8 ± 30.6 and 196.8 ± 96.5 N/mm, which shows a difference from the previous study. It seems that our homogeneous gyroid inner pattern does not mimic diversity in real bone, resulting in relatively high stiffness.

Previously reported synthetic plastic bone models did not consider the cortical bone layer, hardness of the trabecular layer, and its external shape^27^. Cadaver bones vary in shape, mechanical properties, and bone quality or strength^28^. Furthermore, cadavers are not easily available, and it is extremely difficult to obtain osteoporotic cadavers. Therefore, it is difficult to measure the biomechanical properties of implants, such as suture anchors, especially in OBs^9^. Our bone simulators closely replicated actual bone shape and mechanical properties through 3D printing and CT images. This approach offers a valuable means of assessing the mechanical properties of medical devices that are applied to the bones. Moreover, it has the potential for the pre-evaluation of anchor fixation stability in various positions by using patient-specific simulators based on CT images of patients with bone deformities and conducting procedural simulations.

This study has some limitations. First, we considered the actual density and structure of the trabecular layer of OBs and NOBs but did not mimic the trabecular microstructure realistically. Second, the cortical bone of the simulator was mimicked only by controlling the cortical bone layer in OBs. In an OB, the density or porosity of the cortical bone is altered along with the thickness. The density or porosity of the cortical layer was not controlled in our study. Real cortical bone has some irregular microstructures (porosity)^29^. In future studies, we will fabricate a humerus simulator using two or more materials considering bone's anisotropic and heterogeneous and evaluate the mechanical properties of the anchor. Lastly, the 3D-printed simulator used in this study was not compared with osteoporotic and non-osteoporotic cadaver bones.

## Conclusion

In simulated OBs, ASAs demonstrated better biomechanical performance than CAs in terms of stability under cyclic loading and stiffness with similar POSs.

## Data Availability

The datasets generated in this study during the current study are not publicly available because the data used in our study were created based on patient images but are available from the corresponding author on a reasonable request.
